# Effective Chikungunya Virus-like Particle Vaccine Produced in Insect Cells

**DOI:** 10.1371/journal.pntd.0002124

**Published:** 2013-03-14

**Authors:** Stefan W. Metz, Joy Gardner, Corinne Geertsema, Thuy T. Le, Lucas Goh, Just M. Vlak, Andreas Suhrbier, Gorben P. Pijlman

**Affiliations:** 1 Laboratory of Virology, Wageningen University, Wageningen, The Netherlands; 2 Queensland Institute of Medical Research, Brisbane, Queensland, Australia; 3 The University of Queensland, St. Lucia, Queensland, Australia; Centers for Disease Control and Prevention, United States of America

## Abstract

The emerging arthritogenic, mosquito-borne chikungunya virus (CHIKV) causes severe disease in humans and represents a serious public health threat in countries where *Aedes spp* mosquitoes are present. This study describes for the first time the successful production of CHIKV virus-like particles (VLPs) in insect cells using recombinant baculoviruses. This well-established expression system is rapidly scalable to volumes required for epidemic responses and proved well suited for processing of CHIKV glycoproteins and production of enveloped VLPs. Herein we show that a single immunization with 1 µg of non-adjuvanted CHIKV VLPs induced high titer neutralizing antibody responses and provided complete protection against viraemia and joint inflammation upon challenge with the Réunion Island CHIKV strain in an adult wild-type mouse model of CHIKV disease. CHIKV VLPs produced in insect cells using recombinant baculoviruses thus represents as a new, safe, non-replicating and effective vaccine candidate against CHIKV infections.

## Author Summary

Viruses that are transmitted by mosquitoes represent major threats for human health all over the world. One of these viruses is the Chikungunya virus (CHIKV). CHIKV is transmitted by the Asian Tiger mosquito, which is making ground to more temperate regions such as Europe, and thereby increasing the risk of CHIKV infections. The virus causes severe fevers and long lasting joint pains. Unfortunately, there is no vaccine to combat CHIKV infections. This study describes the development of a virus-like particle (VLP) vaccine against CHIKV infections, which is produced in insect cells. VLPs are structurally identical to the wild type virus, but these particles cannot replicate due to the absence of the viral genome. The CHIKV VLPs that were produced using the baculovirus-insect cell expression system, were correctly produced and mimic live CHIKV in structural organisation and protein function. Interestingly, a single administration of a low dose (1 µg/mouse) of non-adjuvanted VLPs induced robust neutralizing antibody titers and provided complete protection upon CHIKV challenge against viraemia and disease symptoms. This new effective, safe and scalable vaccine candidate represents a step forward in the prevention of CHIKV infections.

## Introduction

Chikungunya virus (CHIKV) is a mosquito-borne, single-stranded, positive-sense RNA virus (genus *alphavirus*) that has caused sporadic outbreaks every 2–50 years of predominantly rheumatic disease, primarily in Africa and Asia. CHIKV recently (2004–2011) produced the largest epidemic recorded for an *alphavirus* with an estimated 1.4 to 6 million patients, and imported cases reported in nearly 40 countries including Europe, Japan and the USA. The first autochthonous CHIKV infections in Europe (Italy in 2007 and France in 2010) were also seen during this epidemic. Although *Aedes aegypti* is the traditional vector for CHIKV, the recent outbreak was associated with the emergence of a new clade of CHIKV viruses, which were efficiently transmitted by *Aedes albopictus* mosquitoes, a vector that has seen a dramatic global expansion in its geographic distribution [1], [2]. CHIKV is a biosafety level 3 (BSL3) pathogen and has been declared a Category C Priority Pathogen by the National Institute of Allergy and Infectious Disease (NIAID) in the United States. The US Army has long recognized that CHIKV could be used as a biological weapon [3]. The word “chikungunya” is derived from the Makonde language (Tanzania) and means “that which bends up” referring to the severe joint pain-induced posture of afflicted individuals. CHIKV disease is characterized by acute and chronic polyarthritis/polyarthralgia, which is usually symmetrical and often incapacitating, with other symptoms such a fever, rash, myalgia and/or fatigue often also present during the acute phase. Arthropathy usually progressively resolves over weeks to months, usually without long-term sequelae; however, CHIKV infections can sometimes cause severe disease manifestations and mortality [2], [4].

CHIKV is an enveloped virus of ∼70 nm and has an RNA genome of ∼11,800 bp [5]. Alphaviral RNA encodes two polyproteins; the non-structural polyprotein and the structural polyprotein. The structural polyprotein is translated from a 26S subgenomic mRNA and is processed into the 5 structural proteins; capsid (C), E3, E2, 6K and E1 [6]. The viral RNA is encapsidated in a ∼40 nm nucleocapsid, which is tightly enclosed by a host-derived lipid bilayer envelope displaying the viral envelope glycoproteins E1 and E2. The glycoproteins are arranged in 80 trimeric spikes composed of three assembled E1–E2 heterodimers. The trimeric spikes are essential for budding of new virus particles, host receptor recognition and attachment (via E2), and cell entry via pH-dependent endocytosis (via E1). Upon translation of the structural polyprotein, the capsid protein C is autocatalytically cleaved from the structural polyprotein and encapsidates cytoplasmic viral genomic RNA. The remaining envelope polyprotein (E3E26KE1) is further processed in the endoplasmic reticulum (ER). The resulting membrane bound E3E2 (also known as precursor E2 or PE2) and E1 form heterodimers, with three of these heterodimers assembling to form the trimeric spikes. Prior to surface exposure of the trimeric spikes, PE2 undergoes furin-dependent cleavage to release E3 from the trimeric spike [7], [8], [9].

At present, no licensed vaccine or particularly effective drug is available for human use for any *alphavirus*. A number of pre-clinical CHIKV vaccines have been described, including inactivated virus formulations [10], [11], [12], live-attenuated virus vaccines [13], [14], [15], chimeric virus vaccines [16], DNA vaccines [17], [18], a recombinant adenovirus vaccine [19], subunit protein vaccines [20], [21], [22] and a virus-like particle (VLP) formulation [23]. A formalin-inactivated *alphavirus* vaccine has been shown to be immunogenic in humans [24]. However, growth of large quantities of CHIKV for vaccine manufacture is complicated by the requirement for appropriate BSL3 containment. A live-attenuated CHIKV vaccine (TSI-GSD-218), although immunogenic, in a human phase II study caused side effects including arthralgia [25]. DNA vaccines have so far not been particularly effective at generating antibody responses in humans [26], which is a concern as antibodies are believed to be required for protection against CHIKV infections [12], [27].

VLPs mimic the native virus surface architecture and protein conformation, which often makes them potent inducers of protective antibody responses in the absence of adjuvants [21]. A CHIKV VLP-based vaccine was recently produced by DNA transfection of mammalian cells, and provided protection in both mice and non-human primates [23], [27]. Although this VLP approach is promising, recombinant baculovirus expression systems out-perform systems utilising DNA plasmid transfection in mammalian cells in a number of areas, in particular cost and scalability [28].

Herein we describe the generation and *in vivo* testing of a CHIKV VLP vaccine generated using a recombinant baculovirus-insect cell expression system [29]. Baculovirus expression in insect cells has proven to be a safe and efficient method for producing heterologous proteins for research, diagnostics and vaccine development. Protein expression in insect cells has the benefit of accurate protein folding and post-translational processing of, for instance, complex glycoproteins [30]. Veterinary baculovirus-produced subunits or VLP vaccines have been on the market for many years [30]. The first human baculo-based vaccine, the cervical cancer VLP vaccine (Cervarix, GlaxoSmithKline) received FDA approval in 2007 [31]. A recombinant influenza virus vaccine (FluBlok, Protein Sciences) is currently under final review by the Food and Drug Administration of the USA [32]. These products have paved the way for future licensing of new baculovirus-based pharmaceutical products and/or vaccines. Recombinant baculoviruses have also been used successfully to expressed *alphavirus* proteins [33], [34], *alphavirus* VLPs [35] and functionally active CHIKV subunits [22]. Insect cells are readily adaptable to suspension cultures, making scalability a key benefit of the baculovirus system; insect-cell bioreactors of 2,000 l culture volume are in routine use in industry [36], [37]. Therefore, we believe the CHIKV VLPs described here will make a safe and effective vaccine amenable to large scale and rapid production.

## Methods

### Cells and viruses

Adherent *Spodoptera frugiperda* (*Sf*21)-cells (Invitrogen) were maintained as a monolayer tissue culture in Grace's insect cell medium (Invitrogen), supplemented with 10% foetal bovine serum (FBS, Gibco). *Sf*9-easy titration (ET) cells [38] were maintained as a monolayer cell culture using Sf900II (Invitrogen) serum-free medium (SFM), supplemented with 5% FBS and 200 µg/ml Geneticin (Gibco). Recombinant baculoviruses were generated according to the Bac-to-Bac baculovirus expression system, using an adapted *Autographa californica* nucleopolyhedrovirus (AcMNPVΔp10Δcc) backbone [22], [39]. The cloning fragment of the complete CHIKV-S27 structural polyprotein (Genbank accession # AF369024) was synthetically generated (GeneArt) and equipped with AttB recombination sites to enable Gateway cloning (Invitrogen). The 3842 bp CHIKV fragment was cloned into pDONR207 (Invitrogen) donor plasmid and subsequently transferred to the pFastBacI analogue pDEST8 (Invitrogen). The CHIKV-S27 structural cassette was then recombined into the AcMNPVΔp10Δcc, resulting in Ac-S27. Recombinant baculovirus titers were determined by end point dilution assays using *Sf*9-ET cells and expressed in tissue culture infectious dose 50 (TCID_50_)/ml.

### CHIKV VLP production and purification

To produce CHIKV VLPs, 8×10^6^ *Sf*21-cells were seeded in a 75 cm^2^ culture flask and infected with Ac-S27 at a multiplicity of infection (MOI) of 10 TCID_50_ units per cell. Infections were performed under serum-free conditions on a shaking platform at 27°C and cells were incubated at 27°C for 72 h. Next, cells were separated from the medium fraction by low speed centrifugation. The cell fraction was washed in phosphate buffered saline (PBS) and finally stored in 200 µl PBS at −20°C. Secreted protein fractions were precipitated from the medium with 7% (w/v) polyethylene glycol (PEG)-6000 and 0.5 M NaCl for 2 h at room temperature (RT). Pellets were resuspended in 1 ml GTNE buffer (*200 nM Glycine*, *50 mM Tris/HCl*, *100 mM NaCl*, *1 mM EDTA*, *pH 7.3*) and loaded on a discontinuous 70% (w/v), 40% (w/v) sucrose in GTNE gradient. Sucrose gradients were centrifuged at 27,000 rpm (SW55 rotor, Beckman) for 2 h at 4°C. The 70%-40% interphase band was isolated and resuspended in 5 ml GTNE buffer. The VLPs were pelleted by centrifugation with 30000 rpm, for 30 min at 4°C. The pellet was resuspended in 50 µl GTNE, checked for integrity by transmission electron microscopy and stored at −80°C. VLPs were quantified based on specific E2 protein content, which was determined by Western analysis and via Bradford protein assay (Biorad) and calculated using purified E2 subunit [22] as a reference.

### Protein analysis

Protein expression and processing of infected *Sf*21-cell fractions and purified CHIKV VLP fractions were analysed by sodium dodecyl sulphate polyacrylamide gel electrophoresis (SDS-PAGE) and Coomassie Brilliant Blue (CBB) staining. The purified VLPs and cell fractions were denatured in a gel loading buffer containing SDS and β-mercaptoethanol, incubated for 10 min at 95°C and clarified by centrifugation for 1 min at 14,000 rpm. After electrophoresis, denatured proteins were transferred to an Immobilon membrane (Millipore) for analysis by Western blot (WB). Membranes were blocked in 3% skimmed milk in PBS-0.1% Tween-60 (PBST) for 1 h at RT or overnight (ON) at 4°C. Blocked membranes were washed 3×5 min with PBST and subsequently incubated for 1 h at RT with rabbit polyclonal anti-E1 and anti-E2 [22], 1∶15,000 and 1∶20,000 diluted in PBST, respectively. Membranes were washed and treated with alkaline phosphatase (AP) conjugated, goat anti-rabbit IgG monoclonal antibodies (Sigma), 1∶3000 times diluted in PBST, for 45 min at RT. Membranes were washed 2×5 min with PBST and 1×10 min with AP-buffer (*100 mM NaCl*, *5 mM MgCl_2_*, *100 mM Tris-HCl*, *0.1% Tween 20*, *pH 9.5*). Proteins were detected by NBT/BCIP staining (Roche).

### PNGase F treatment

Infected cell- and medium-fractions were treated with PNGase F (New England Biolabs) to determine the glycosylation status of the CHIKV glycoproteins E1 and E2. Protein samples were treated with 1 µl denaturing buffer in 9 µl MilliQ for 10 min at 95°C. The denatured proteins were subsequently incubated with 2 µl G7 reaction buffer, 2 µl 10% NP40 buffer, 0.5 µl PNGase F in 4.5 µl MilliQ for 1 h at 37°C. Treated and non-treated protein samples were analyzed by SDS-PAGE and WB.

### Syncytia induction assay

*Sf*9-ET cells [38] were infected with Ac-S27 and Ac-GFP with a MOI of 10 TCID_50_/ml in Sf900-II SFM medium. A recombinant AcMNPV expressing CHIKV 6KE1 (Ac-6KE1) [22] was used as a positive control. The medium (pH = 6.4) was supplemented with 0.2 mg/ml cholesterol (Sigma) as described previously [22]. Cell fusion was induced 72 hpi, by treating the cells for 2 min with acidified medium with pH = 5.8, pH = 5.5 and pH = 5.0, respectively. Syncytia formation was analyzed 4 h post treatment by fluorescence light microscopy.

### Immunofluorescence assay

To determine surface expression of CHIKV-E1 and -E2, *Sf*21-cells were infected with Ac-S27, with a MOI of 10 TCID_50_/ml in Grace's insect SFM (Invitrogen). Cells were harvested 72 hpi and washed with PBS. Next, cells were incubated with PBS containing 1∶5000 diluted rabbit α-E1 and rabbit α-E2 polyclonal antibodies, for 1 h at RT. Cells were washed 3×5 min with PBS and treated with 1∶1000 diluted goat-anti-rabbit Alexa fluor 488 (Invitrogen) for 1 h at RT. Finally, cells were washed and treated with 1∶100 diluted Hoechst stain for 5 min at RT. Cells were analyzed using fluorescence light microscopy.

### Time course expression assay and VLP- ELISA

To analyze CHIKV-VLP production in time, *Sf*21 cells were infected with Ac-S27 and samples were taken at intermediate time points from 4 h to 69 hpi. The medium fraction was analyzed for the amount of VLPs in triplo by enzyme linked immunosorbent assay (ELISA). ELISA plates (Greiner Bio-One) were coated with 2.5 µg/ml rabbit α-E2 polyclonal antibodies [22] in coating buffer for 2 h at RT. Plates were washed three times in PBST, and medium samples were loaded for 2 h at 37°C. The plates were washed three times and treated with 1∶500 diluted α-E2 monoclonal antibodies (52B2, provided by Lucas Goh) for 2 h at 37°C. Plates were washed and incubated for 2 h at 37°C with 1∶500 diluted, AP-conjugated, goat-anti-mouse IgG monoclonal antibodies (Sigma). Finally, plates were washed three times and treated with 1 mg/ml phosphatase substrate (Sigma) in substrate buffer for 45 min at 37°C. Absorbance was measured at 405 nm using a FLUOstar Optima (BMG Labtech).

### Electron microscopy

Copper 400 square mesh grids (Veco) were treated by Argon gas discharge and loaded with 10 µl sample for 2 min at RT. Excess liquid was removed and the grids were washed five times with MilliQ. Finally, grids were treated with 2% uranyl acetate for 15 s, excess uranyl acetate was carefully removed using filter paper. The grids were air dried and analyzed with a JEOL JEM 1011 transmission electron microscope.

### Vaccination

The purified CHIKV VLPs were used as a vaccine. Female C57/BL6 mice (6–12 weeks old) were vaccinated once subcutaneously on the back above the base of the tail with 0.1 µg VLPs or 1 µg VLPs in 50 µl RPMI 1640 medium (Gibco). As negative control, a purified fraction of Ac-GFP infected culture media was used. Binary ethylenimine (BEI)-inactivated purified CHIKV was used as positive control and produced as described [12]. Where indicated, the VLP suspension was formulated with 10 µg/mouse Quil A (Iscotec) prior to injection.

### Murine virus neutralization and ELISA antibody assays

The neutralizing ability of the mouse serum was assayed as described [19]. Serum from each mouse was heat-inactivated at 56°C for 30 min and serially diluted in a 96-well plate. Diluted serum was incubated with 200 TCID_50_/ml CHIKV of the Réunion Isalnd CHIKV isolate for 2 h at 37°C. Vero cells (10^4^/well) were added to the plate and incubated for 5 days at 37°C. The serum dilution yielding >95% protection against CPE was determined by staining the cells with crystal violet. Determination of the CHIKV-specific IgG1 and IgG2c antibody titers was performed by ELISA as described previously [12].

### Viral challenge and disease monitoring

Female C57BL/6 mice (6 to 12 weeks old) were inoculated with CHIKV (LR2006-OPY1), and viraemia and foot swelling were determined as described previously [12], [40]. Foot swelling was monitored by measuring the height and width of the metatarsal area of the hind feet using digital callipers and is presented as a group average of the percentage increase in foot height times width for each foot compared with the same foot on day 0. All animal experiments were approved by the Queensland Institute of Medical Research (QIMR) animal ethics committee and adhered to the Australian code of practice for the care and use of animals for scientific purposes (NHRMC, Australia; 7th edition 2004).

### Statistical analysis

Statistical analysis on antibody responses was performed using SAS, specifically one way ANOVA with Tukey post-hoc test. Statistical analysis on viraemia and foot swelling was performed using IBM SPSS Statistics 19. For comparison of two samples, the t-test was used when the difference in the variances was less than 4 and skewness was greater than minus 2 and kurtosis was less than 2; otherwise, a non parametric test was used, specifically, Mann-Whitney U test if variance was less than 4 or Kolmogorov Smirnov test if greater than 4.

## Results

### Expression analysis of the CHIKV structural polyprotein, produced in insect cells by recombinant baculoviruses

**Figure 1:**
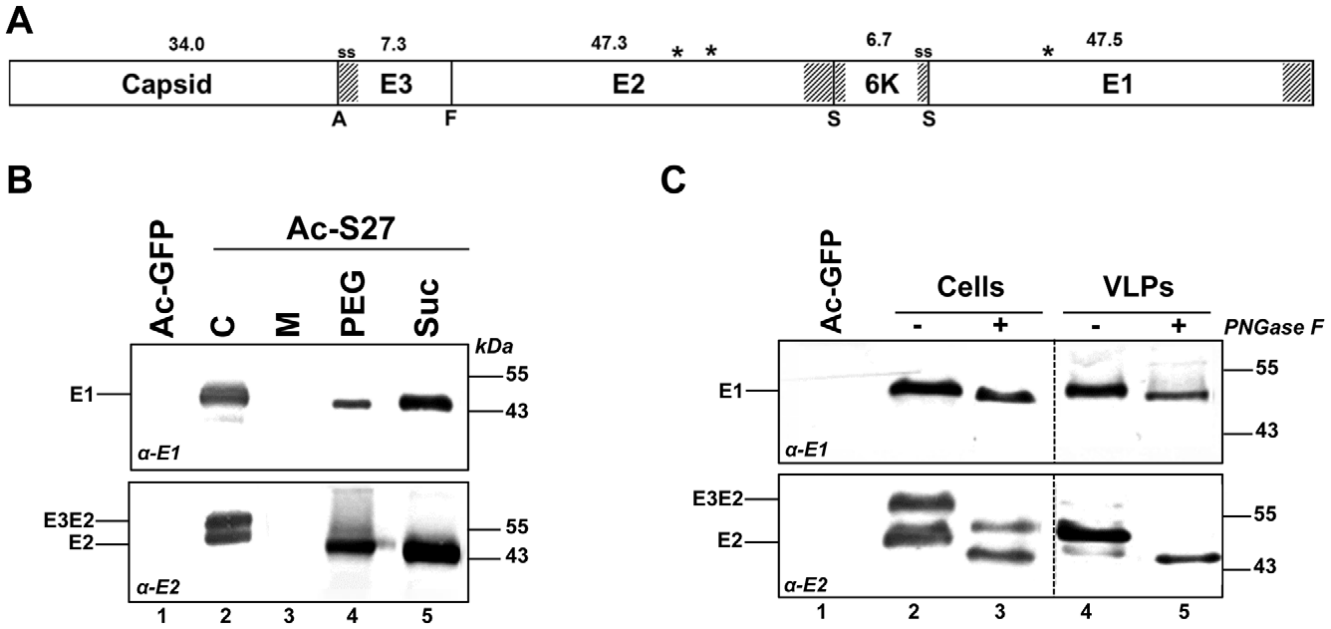
CHIKV structural cassette expression and VLP production, using recombinant baculoviruses. A) Schematic representation of the CHIKV structural cassette, as it was expressed in insect cells. Shaded areas represent transmembrane domain and ss signifies signal sequences. A, F and S indicate autocatalytic, furin and signalase cleavage sites, respectively. Asterisks indicate N-glycosylation sites and the molecular mass (in kDa) of the proteins is depicted. B) CHIKV E1 and E2 expression in the *Sf*21 cell (C) and medium (M) fraction was analysed by WB using α-E1 and α-E2 polyclonal antibodies. VLPs were precipitated using PEG-6000 (PEG) and subsequently purified using discontinuous sucrose gradient centrifugation (Suc). C) The glycosylation status of CHIKV E1 and E2 was analysed by WB after PNGase F treatment. Ac-GFP was used as a negative control.

A recombinant baculovirus (Ac-S27) was generated to produce CHIKV VLPs by expressing the complete CHIKV-S27 structural polyprotein (C, E3, E2, 6K, E1) (**Fig. 1A**). The coding sequence of the structural polyprotein was cloned downstream the polyhedrin promoter in an AcMNPV backbone, after which *Sf*21 cells were infected with a MOI = 10 TCID_50_/ml. Glycoprotein expression in the cell fraction as well as in the medium fraction was analyzed by WB using α-E1 (**Fig. 1B**) and α-E2 (**Fig. 1C**) polyclonal antibodies. A recombinant baculovirus expressing GFP (Ac-GFP) was used a negative control. Western analysis of the cell fraction yielded protein bands of ∼50 kDa for α-E1 (**Fig. 1B** **, lane 2**) and two bands of ∼50 kDa and ∼57 kDa for α-E2 (**Fig. 1C** **, lane 2**). These sizes correspond to predicted molecular masses of E1 (47.5 kDa), E2 (47.3 kDa) and its precursor E3E2 (54.6 kDa), respectively. CHIKV glycoproteins were also detected in the medium fractions after PEG-precipitation (**Fig. 1B and 1C** **, lane 4**). The molecular mass of observed protein bands corresponds to mature E1 and E2. The PEG-precipitated medium fraction containing the VLPs was subjected to discontinuous sucrose gradient purification. The 70% - 40% intermediate phase was isolated and analyzed on WB (**Fig. 1B** **, lane 5**). This resulted in a further concentration of CHIKV- E1 and E2. In addition, a ∼30 kDa protein band, corresponding to the predicted molecular weight of CHIKV-C, was readily observed on the coomassie-stained SDS-PAGE gel (not shown), suggesting that the VLPs contain a nucleocapsid. To analyze the glycosylation status of the glycoproteins E1 and E2, the infected cell fraction and the purified VLPs were treated with PNGase F, which enzymatically removes glycan residues from N-glycosylated proteins fractions. CHIKV-E1 is predicted to be N-glycosylated at N141, whereas E2 is predicted to be N-glycosylated at N263 and N273 (**Fig. 1A**) [41]. PNGase F treatment resulted in an expected reduction in molecular mass of CHIKV-E1 in both the cell fraction and purified VLPs (**Fig. 1C** **, lane 2–5**), indicating that E1 was efficiently N-glycosylated by the insect cells. The size difference between non-treated and treated samples was significantly larger for E2 than that of E1, which may suggest that E2 is N-glycosylated at the two predicted loci (**Fig. 1C** **, lane 2–4**). In addition, both protein bands (presumably E2 and E3E2, based upon earlier observations [22]) in the double-band pattern that were found using α-E2 detection (**Fig. 1B and 1C**), appeared to be fully glycosylated.

### CHIKV glycoproteins E1 and E2 localize to the surface of *Sf*-cells

**Figure 2:**
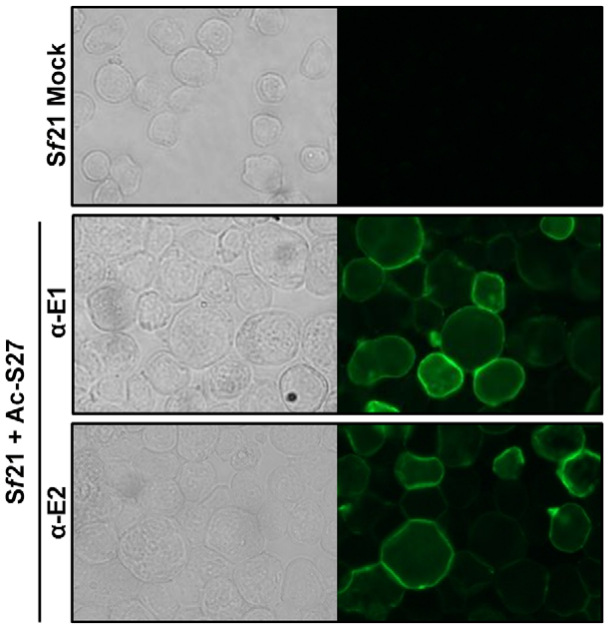
CHIKV-E1 and –E2 detection on the surface of Ac-S27 infected *Sf*21 cells. Sf21 cells were infected with Ac-S27 and subjected to immunostaining using α-E1 and α-E2 antibodies. Cells were analysed by fluorescent microscopy where positive staining indicates E1 or E2 surface exposure.

During natural infections, CHIKV-E1 and -E2 are assembled into trimeric spikes, which are expressed at the surface of the host cell, to enable budding of new virus particles. Expression analysis has made clear that E1 and E2 are both glycosylated and that a fraction of PE2 is processed by furin. To analyze whether the glycoproteins were subsequently translocated to the cell plasma membrane, non-permeable *Sf*21 cells infected with Ac-S27 were treated with α-E1 and α-E2 to enable immunofluorescence analysis. Treated cells displayed ring-like structures (**Fig. 2** **, middle and bottom**), indicating that the glycoproteins are indeed exposed at the surface of the infected cells. The non-infected mock cells did not reveal these ring-like structures (**Fig. 2** **, top**).

### CHIKV E1 retains fusogenic activity when expressed by recombinant baculoviruses

So far, experiments have shown that CHIKV E1 and E2 are expressed, processed correctly and exposed at the surface of the infected host cell. This suggests that maturation of the recombinant CHIKV structural proteins appears to correspond to what happens during natural virus infection. To test whether CHIKV E1 retains its functionality as a fusion protein, a pH-dependent syncytia formation assay was performed. *Sf*9-ET cells were infected with Ac-S27, Ac-6KE1 and Ac-GFP (negative control). Ac-6KE1 is a recombinant baculovirus that expresses individual fusogenic E1 [22] and was used as a positive control. Infected cells were treated for 2 min with acidified medium (pH = 5.8, pH = 5.5 and pH = 5.0) and screened for syncytia formation 4 h post treatment.

**Figure 3:**
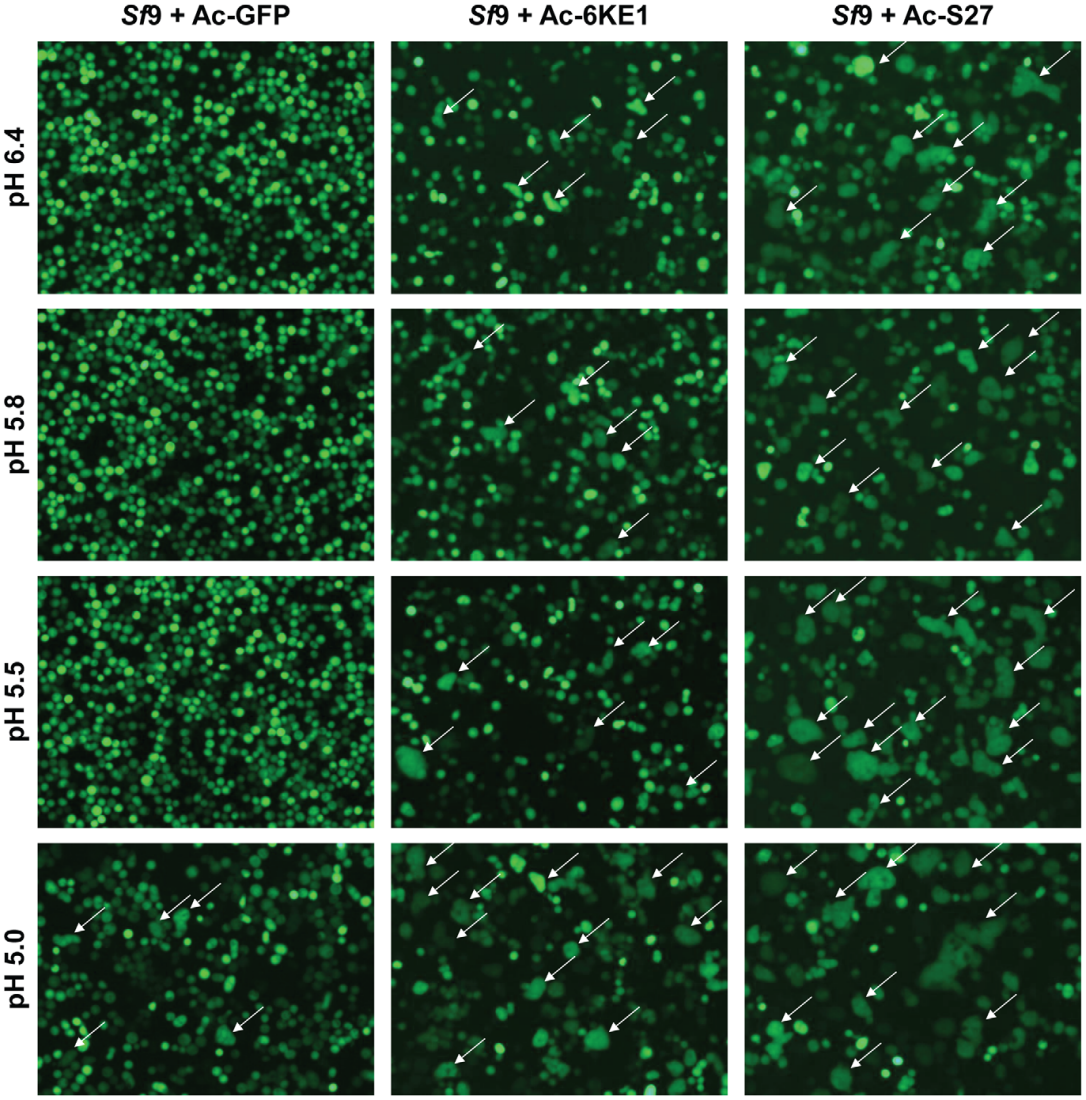
Syncytia formation assay on Ac-S27 infected insect cells. *Sf*9 ET- cells were infected with Ac-S27 and treated 72 hpi with acidified culture medium of pH = 6.4, 5.8, 5.5 and 5.0 for 2 min. Cells were analysed 4 h post induction and syncytia are indicated with white arrows.

Syncytia formation at pH = 6.4, pH = 5.8, pH = 5.5 was readily observed in cells expressing CHIKV structural proteins (Ac-S27) (**Fig. 3** **, right**) and in the positive control (Ac-6KE1) (**Fig. 3** **, middle**) but not in the negative control (Ac-GFP) (**Fig. 3** **, left**). Syncytia observed in Ac-S27 infected cells were slightly more abundant and also larger in size, suggesting that E1 in its native conformation, i.e. in trimeric spikes closely associated with E2, displays increased fusogenic activity as compared to individual E1 expression in Ac-6KE1 infected cells. In contrast to Ac-S27 and Ac-6KE1, Ac-GFP infected cells were only able to fuse when treated with acidified medium of pH = 5.0 (**Fig. 3** **, left**). This was due to the pH-dependent activity of the baculovirus fusion protein GP64, which becomes active only at values below pH = 5 [42]. The formation of syncytia correlates with the presence of E1 on the surface, individually expressed or expressed as a part of the CHIKV structural polyprotein. Therefore, it can be concluded that E1 retains its fusogenic properties when expressed in *Sf*-cells.

### Production of CHIKV virus-like particles in insect cells

**Figure 4:**
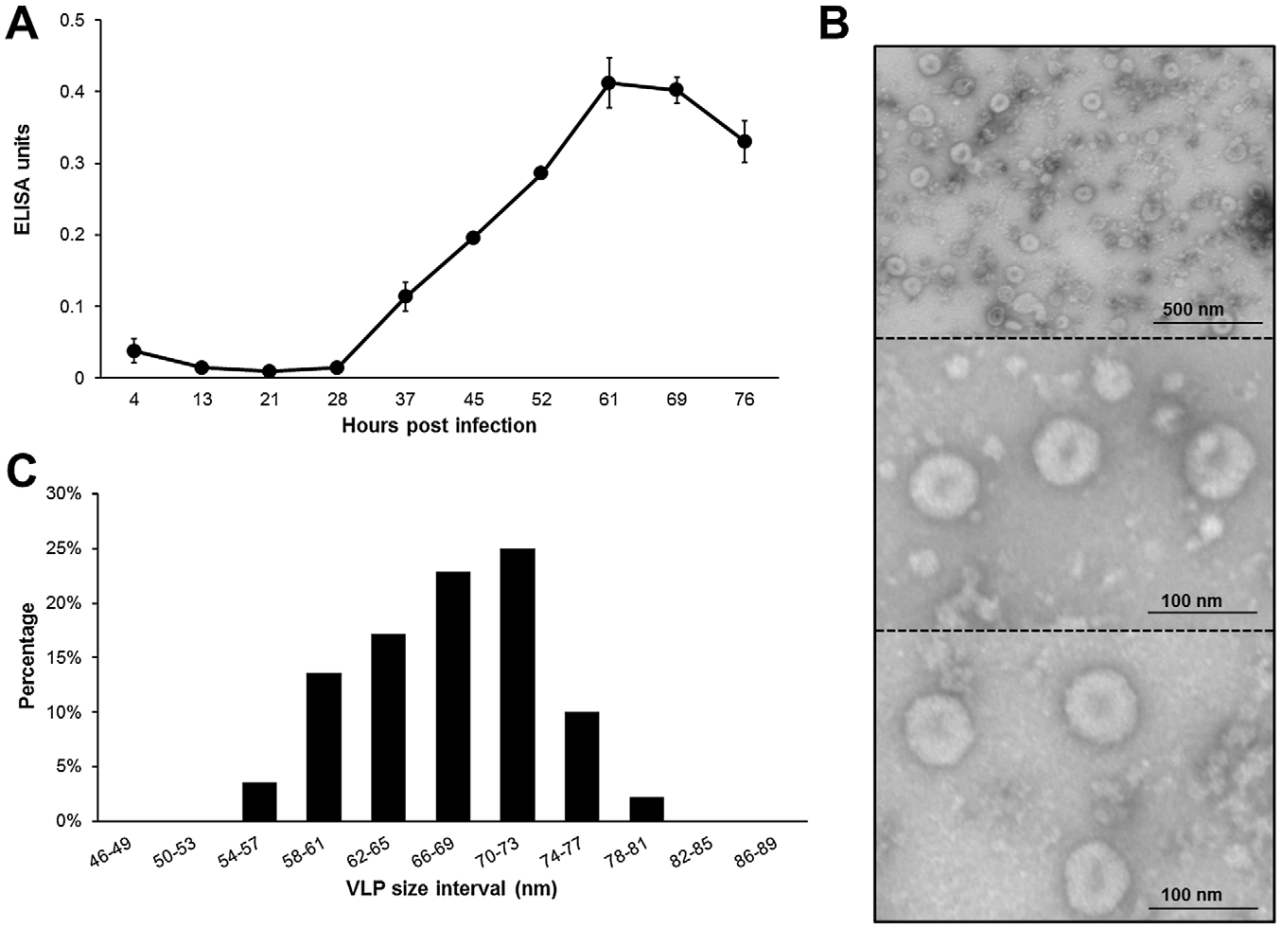
CHIKV VLP production in *Sf*21 insect cells. A) *Sf*21 cells were infected with Ac-S27 in duplo and VLP production was followed over time. Medium fraction samples were taken at the indicated time points and analysed on E2 content by sandwich ELISA. B) Electron micrographs of CHIKV-VLPs at a 12,000∶1 (top) and 40,000∶1 (middle and bottom) magnification. The medium fraction of infected *Sf*21 cells was analysed by transmission electron microscopy (TEM) for the presence of VLPs. C) The size of 200 VLPs were determined using TEM to determine the relative VLP size range.

Baculovirus expression of the complete CHIKV structural cassette in insect cells leads to correct processing of the CHIKV glycoproteins E1 and E2, which both are exposed on the surface of the host cell. Western analysis of the medium fraction indicated that both E1 and E2 are present in the medium and can be concentrated by discontinuous sucrose gradient purification. These findings strongly suggest that VLPs were formed and were secreted in the medium. To analyze VLP production in time, a time-course expression assay was performed and CHIKV-VLPs were detected by a sandwich-ELISA, using α-E2 antibodies (**Fig. 4A**). *Sf*21-cells were infected in duplo with Ac-S27 and medium samples were taken within 7 to 9 h intervals. VLP production in *Sf*21-cells initiated at ∼28 hpi, typical for polyhedrin expression. Production peaked at ∼61 hpi and longer production periods, yielded lower amounts of VLPs probably due to cell death. For the final verification of CHIKV-VLP production, insect cells were infected with Ac-S27 and the medium fraction was subjected to discontinuous sucrose gradient purification. The isolated VLP fraction was analyzed by transmission electron microscopy (TEM) (**Fig. 4B**). Spherical, enveloped particles of ∼65–70 nm, were detected in large numbers and were absent in control infections with Ac-GFP. The isolated VLPs varied in size, within a range of ∼55–80 nm. The diameters of 200 VLPs were measured to determine a relative size distribution (**Fig. 4C**). The CHIKV VLPs fitted a size of 68±14 nm, which is consistent with the reported size (65–70 nm) of *alphavirus* virions. The specific E2 protein content of the isolated VLPs was determined at 40 mg/l.

### Neutralizing antibody and antibody titers following vaccination

**Figure 5:**
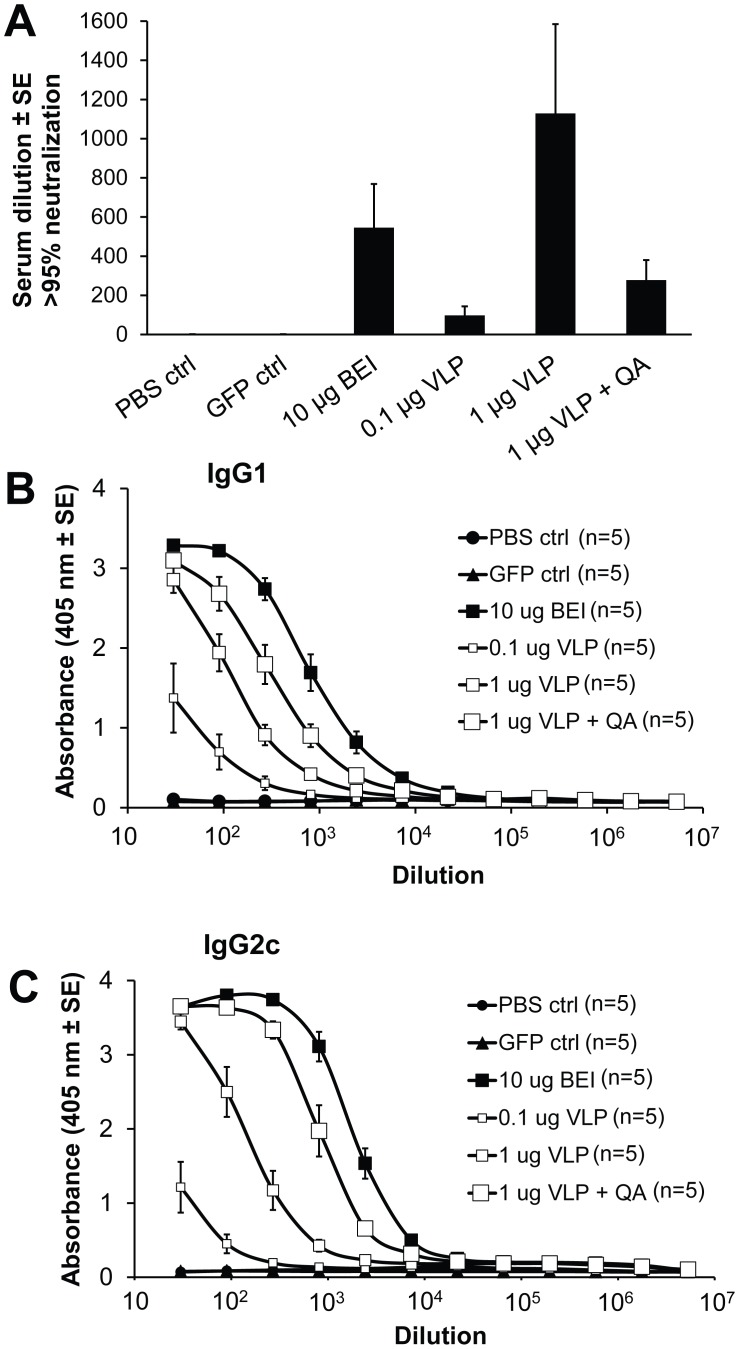
CHIKV neutralization and IgG-isotyping. A) Serum of immunized mice were collected and tested for their neutralizing ability based on >95% protection against CHIKV induced CPE in Vero cells. BEI corresponds to the inactivated CHIKV positive control. B–C) Immunoglobulin-G1 and -2c isotypes were determined using ELISA upon serial dilution. Statistical analysis shows that all CHIKV antigen immunized groups are significantly different from the control groups.

To assess the immunogenicity of the CHIKV-VLPs, C57/BL6 mice were vaccinated once with 0.1 µg or 1 µg of the VLPs, 1 µg of the VLPs formulated with Quil A adjuvant or 10 µg inactivated CHIKV (positive control). The negative controls were PBS and a GFP control. For the GFP control, *Sf*21-cells were infected with Ac-GFP and the infection medium was treated under exactly the same conditions as the CHIKV VLPs. All groups receiving CHIKV antigens generated neutralizing antibody titers that are significantly different (P<0.01) from both control groups (PBS and Ac-GFP) but not significantly different from one another (**Fig. 5A**). The 1 µg VLP dose induced neutralizing antibody titers comparable with those observed after vaccination with 10 µg of inactivated CHIKV (**Fig. 5A**).

CHIKV-specific IgG1 and IgG2c titers were determined by ELISA, and broadly similar titers were seen for IgG1 and IgG2c (**Fig. 5B, C**), which contrasts with natural CHIKV infection where IgG2c dominates [12]. At the 1 µg dose, VLPs produced ∼10–20 fold, significantly lower antibody titers (IgG1 P<0.05, IgG2c P<0.01) than 10 µg inactivated CHIKV. The 0.1 µg VLP dose showed a further ∼10–20 fold reduction in antibody titers compared to 10 µg inactivated CHIKV (IgG1 P<0.01, IgG2c P<0.01) (**Fig. 5B, C**). The addition of Quil A significantly (P<0.05) increased IgG2c but not significantly IgG1 titers by ∼5–10 fold (**Fig. 5B–C**). In summary, a single 1 µg dose of insect-cell derived CHIKV VLPs induced potent CHIKV-specific neutralizing antibody and IgG titers.

### CHIKV challenge of vaccinated C57BL/6 mice

**Figure 6:**
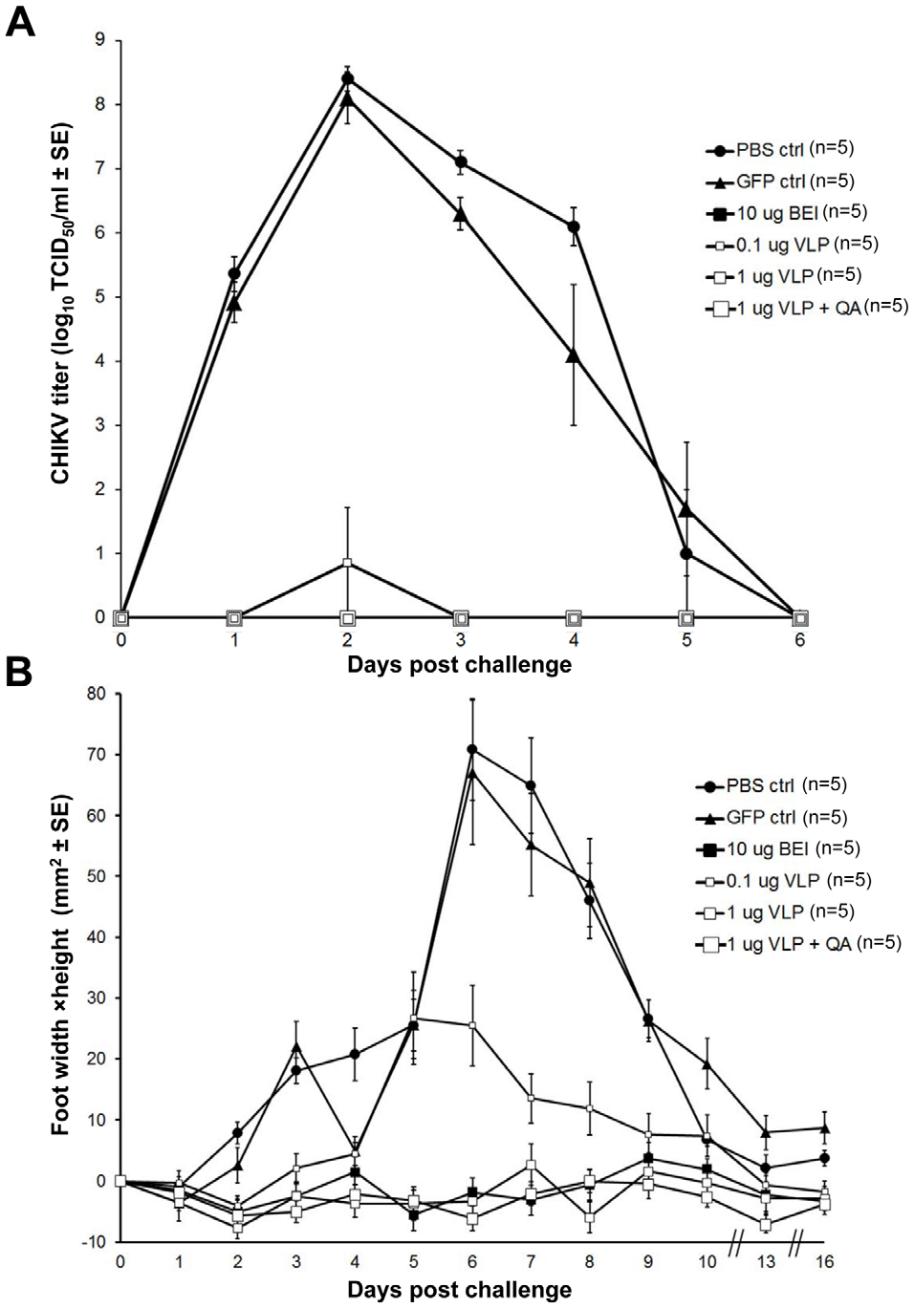
CHIKV VLP vaccination and CHIKV challenge. A) At least 6 weeks old mice were vaccinated with 0.1 µg VLPs, 1 µg VLPs and 1 µg VLPs adjuvanted with Quil A (QA). PBS and GFP were used as a negative control and inactivated CHIKV virus as a positive control (n = 6 per group). Mice were challenged with the Rèunion Island isolate 5 w post infection. Viraemia levels were determined over 6 days. B) Foot size in mm^2^ (width×height) was determined over 16 days post challenge. Statistical analysis shows that mice vaccinated with inactivated CHIKV virus, 1 µg VLPs and 1 µg VLPs adjuvanted with Quil A display significant lack of viraemia or lack of footswelling at times of peak viraemia (1–3 dpi) and peak foot swelling (6–8 dpi) in the control groups, respectively.

Vaccinated mice were challenged 6 weeks post-vaccination with a Réunion Island CHIKV isolate using a recently developed adult wild-type mouse model of CHIKV viraemia and arthritis [12]. PBS-vaccinated animals and animals vaccinated with the GFP control group showed similar viraemia; >10^8^ TCID_50_/ml at 2 d post challenge (**Fig. 6A**). All animals vaccinated with 1 µg VLPs were completely protected against viraemia (**Fig. 6A**). Mice vaccinated with 0.1 µg VLPs, showed a ∼7 log reduction in viraemia on day 2, with virus undetectable on the other days (**Fig. 6A**). Vaccination with 10 µg of inactivated virus also provided complete protection against viraemia as described previously [12]. Arthritis in this model is readily determined by measuring foot swelling and, as expected [12], the inactivated virus completely protected against foot swelling, whereas animals given PBS showed a mean 60–70% increase in foot swelling (**Fig. 6B**). The 1 µg dose of VLPs (with or without Quil A) provided complete protection against foot swelling, while the 0.1 µg dose reduced the peak foot swelling from 60–70% to 20–30% (**Fig. 6B**). These results illustrate that non-adjuvanted VLPs can provide complete protection against CHIKV-induced arthritis.

## Discussion

In this study, recombinant baculoviruses were used to produce CHIKV VLPs in Sf21 insect cells. The VLP proteins were correctly processed and the VLPs provided complete protection against CHIKV viraemia and arthritic disease in a mouse model after a single dose of CHIKV VLPs.

The complete CHIKV structural cassette was cloned downstream the strong polyhedrin promoter of the AcMNPV baculovirus (Ac-S27). Western analysis on cell and medium fractions of *Sf*21-cells that were infected with Ac-S27 indicated that glycoprotein processing (glycosylation, furin cleavage and surface localization) is efficient and that the functionality of E1 as fusion protein is retained. Western analysis on the precipitated medium fraction and purified VLPs shows that only fully matured and glycosylated E2 was incorporated into the VLPs, which suggest that the recombinant VLPs are homogenous. This contrasts with previous findings that uncleaved E3E2 is present in progeny *alphavirus* particles [43], [44]. However, both immature (presumably E3E2) and mature, furin-cleaved E2 fractions were found intracellularly, most likely a result of the very high expression levels of the CHIKV proteins. Alphaviral processing intermediates are commonly found in many different expression systems, including recombinant baculoviruses [33], [34], [45], [46]. The triple-banded E2 pattern previously observed upon individual expression of E3E2 [22] was not found after expression using Ac-S27, indicating that in this case all glycoproteins were efficiently glycosylated. The postulated number of N-glycosylation sites of E1 (n = 1) and E2 (n = 2) correspond to the protein size shifts after PNGase F treatment. These results demonstrate that the processing efficiency of E3E2 in insect cells increases when CHIKV glycoproteins E1 and E3E2 are co-expressed as part of a polyprotein, when compared with expression of E3E2 by itself [22]. To obtain more insight in the exact number and type of glycan-moieties on both E1 and E2, in-depth studies are required (e.g. mass spectrometry), which can precisely indicate to what extent the glycoprotein intermediates are processed.

In addition to correct processing, CHIKV-E1 and E2 were found exposed on the surface of the infected insect cells. The green fluorescent rings found after immunostaining Ac-S27 infected insect cells (**Figure 2**), mark the final stage of processing and translocation of the glycoproteins within the host cell, just prior to the budding of the VLPs. In addition, the retained fusogenic function of E1 was shown by treatment of Ac-6KE1, Ac-GFP and Ac-S27-infected *Sf*9-ET cells with acidified culture medium (pH = 5.8, 5.5 and 5.0) resulting in increased syncytia formation. This was not a consequence of the baculovirus GP64 fusion protein, as the control baculovirus Ac-GFP only formed syncytia at pH = 5.0 [42].

The VLPs were efficiently isolated using discontinuous sucrose gradient purification. Transmission electron microscopy (TEM) analysis revealed that the VLPs were morphologically similar to CHIKV and other *alphavirus* VLPs and that they had a similar diameter of 68±14 nm [23], [35]. The overall baculovirus CHIKV VLP yield (40 mg/L) appeared to be higher than in another study, in which VLPs were produced by DNA-transfection of 293F cells (10–20 mg/L) [23]. This underscores one of the major advantages of the baculovirus-insect cell system for production of recombinant proteins. A further gain in VLP yield is expected in an optimized large-scale insect-cell bioreactor configuration [37].

We show herein that a single vaccination with 1 µg of unadjuvanted CHIKV VLP vaccine was able to completely protect mice from CHIKV-induced viraemia and arthritis in an adult wild-type model of CHIKV arthritis that recapitulates many aspects of the rheumatic human disease, i.e. self-limiting arthritis (joint inflammation), tenosynovitus and myositis [12]. The value of adjuvants in this system remains to be fully explored, with different adjuvants, adjuvant doses and VLP∶adjuvant ratios and their effect on protection needing to be analyzed. The importance of protection studies is highlighted by the ability of Quil A to increase CHIKV-specific IgG titers, but reduce neutralizing titers. A recent human trial also illustrated that aluminum hydroxide adjuvant provided increased immunogenicity for an inactivated Ross River vaccine [47]. The VLPs induced a balanced IgG1/IgG2c response, in contrast with CHIKV infection where IgG2c responses dominate [12]. Whether this would have an important effect on protection is unclear. However, we have recently found that there was no difference in viraemia or arthritis following CHIKV infection of mice deficient in the Fc receptor common gamma chain (unpublished data), suggesting the distinct Fcγ receptor-binding properties of the different antibody isotypes [48] does not play a major role in protection (at least in mice). Interestingly, the 1 µg non-adjuvanted VLP vaccine elicited a similar neutralizing antibody response as compared to 10 µg of inactivated virus (Fig. 5A), yet induced lower IgG2c titers (Fig. 5C). This might suggest that chemical inactivation results in generation of antibodies with reduced neutralizing activity, although further experimentation would be required to confirm this.

Previous vaccination studies using CHIKV VLPs have shown that VLP provided protection in mice and non-human primates against CHIKV infection [23]. In those experiments, two immunizations of 19 µg VLPs with adjuvant were needed to induce protection in mice, while our data show that a single vaccination of 1 µg of non-adjuvanted VLPs induces complete protection against viraemia and foot swelling. Although a different CHIKV strain (West-African isotype, also known as strain 37997) was used to produce VLPs in 293F cells [23], the baculovirus-insect cell expression system may also provide VLPs with better immunogenicity. Glycosylation patterns in insect cells differ from those in mammalian cells, in that lepidopteran insect cells do not process N-glycans to terminally sialylated complex-structures. Differences in glycan processing have been shown to influence glycoprotein immunogenicity [49], [50], [51].

Four different CHIKV strains have been described so far, including the East-, Central- and South African (ECSA) strain, the West-African strain, the Asian strain and the recent Réunion Island strain. The produced VLPs are of the ECSA strain, while the immunized mice were challenged with the Réunion Island strain. Even though these strains belong to the same ECSA phylogroup, it has been shown that ECSA strain based vaccines are able to cross-neutralize against other CHIKV strains and even other alphaviruses from the same serogroup [12], [14], [19]. Therefore, our recombinant CHIKV VLPs would be expected to provide protection against most, if not all, CHIKV strains.

The favorable properties of the recombinant baculovirus-insect cell expression system renders it extremely powerful in the production of subunit or VLP based vaccines. Even though baculoviral replication is lytic to insect cells and heterologous protein production is therefore not continuous, the sheer expression levels reached are high, if not the highest, of all eukaryotic expression systems [28]. Baculovirus expression vectors can be quickly generated and therefore this system is ideally suited for generating emergency vaccines (“pandemic preparedness”) [52]. In addition, insect cells can easily be scaled up in serum-free suspension culture to large culture volumes [53]. Conditions for optimal stability of VLPs produced under serum-free conditions should be determined when this vaccine candidate is further developed by the industry, but so far we have no indications that storage at −80°C is detrimental to VLP integrity.

In conclusion, we have shown that complex structures such as CHIKV VLPs are produced at high levels and were efficiently processed and glycosylated in insect cells using recombinant baculoviruses. More importantly, this is the first study that shows that a single low-dose immunization with 1 µg of non-adjuvanted CHIKV VLPs provided complete protection against viraemia and foot swelling caused by CHIKV infection. We propose CHIKV VLPs produced by insect cells using recombinant baculoviruses to be further developed as a safe and effective vaccine candidate to protect humans against CHIKV outbreaks.
